# Association Between Spinopelvic Parameters and Clinical Outcomes of Patients Treated With Transforaminal Platelet-Rich Plasma Injections for Lumbar Disc Prolapse

**DOI:** 10.7759/cureus.66995

**Published:** 2024-08-16

**Authors:** Navin Balaji R, Prabhu Ethiraj, Manoj K Ramachandraiah, Arun Kumaar, Nagakumar J S

**Affiliations:** 1 Department of Orthopaedics, Sri Devaraj Urs Medical College, Sri Devaraj Urs Academy of Higher Education and Research, Kolar, IND

**Keywords:** pelvic tilt, pelvic incidence, platelet rich plasma, spinopelvic parameters, lumbar disc prolapse

## Abstract

Introduction

Lumbar disc prolapse mainly occurs in the regions involving the L4-L5 and L5-S1 vertebrae. In this context, the significance of spinopelvic parameters becomes notably prominent. Non-invasive management of lumbar disc prolapse encompasses medicinal therapy, physical therapy, exercise, and epidural injections. Because of its autologous blood origin, platelet-rich plasma (PRP) therapy has minimal risks associated with immunogenic reactions and side consequences. We evaluated the extent of pain reduction and improvement of functional outcomes in patients having discogenic low back pain with modified spinopelvic parameters before and after undergoing transforaminal autologous PRP injection interventions.

Methodology

An observational study was conducted between September 2022 and August 2023 on 100 patients with low back pain for six months. The study population comprised patients who did not respond to conservative treatment; they were recruited from the orthopaedic ward of the emergency medicine department and outpatient department at the RL Jalappa Hospital and Research Centre affiliated with Sri Devaraj Urs Medical College in Kolar, India. Every patient received a thorough evaluation that comprised an extensive medical history, a clinical examination, and imaging of the lumbosacral spine from both the front and side views. After obtaining the patient's consent and confirming their readiness for surgery, a PRP injection was administered. The injection technique followed the standardized protocol and was performed by an experienced spine surgeon in collaboration with orthopaedic residents. Pain evaluations utilizing the Visual Analog Scale (VAS) and assessment of functional outcomes using the Oswestry Disability Index (ODI) scale were conducted before and after PRP injections at the one- and six-month follow-ups.

Results

The average age of the study participants was 41.82 ± 5.0 years, with 55% of them being male. A total of 39% of study samples exhibited an increased angle in spinopelvic parameters. The independent t-test revealed a statistically significant difference in the mean score of back pain, limb pain, and ODI score between patients with increased and decreased angles of spinopelvic parameters before and after injection (p=0.0001). The severity of back pain, leg pain, and functional disability was significantly reduced in patients with increased angles of spinopelvic parameters following PRP injections at the one-month follow-up (p=0.0001). However, at the six-month follow-up, patients encountered recurring symptoms and worsening back pain, leg pain, and functional disability compared to the one-month follow-up. Conversely, the severity of back pain, leg pain, and functional disability has been markedly reduced in the patients with lower spinopelvic parameter angles following PRP injections at one month (p=0.0001) and six months (p=0.0001) compared to pre-injections.

Conclusion

During the long-term follow-up, subjects with elevated spinopelvic parameter angles reported a lower level of improvement in functional outcome, leg pain, and back pain. The impact of spinopelvic parameters on back pain severity and functional disability is substantial, significantly affecting the functional outcome of patients with lumbar disc prolapse.

## Introduction

Non-specific low back pain is a common condition that affects people of all ages and is a major cause of absenteeism worldwide, with a lifetime prevalence rate of approximately 30%. Lumbar disc prolapse is one of the major causes of this sort of back pain [1]. "Spinopelvic balance" is a term used to describe the synchronized movement of the pelvis and lumbar spine, which is vital for maintaining proper posture. Lumbar lordosis (LL) is one of the spinopelvic parameters defined by the segments involving the L4-5 and L5-S1 vertebrae. These segments play a significant part in the entire process. The relevance of spinopelvic parameters becomes especially obvious when one considers the fact that lumbar disc prolapse primarily occurs in these segments [2].

Standing lateral radiographs taken with the knees and hips completely extended provide the most precise assessment of the sacropelvic orientation, which is defined by how the patient is positioned in space [3]. When it comes to pelvic structure and alignment, pelvic incidence (PI), pelvic tilt (PT), and sacral slope (SS) are frequently used as defining characteristics [3].

Quantitative evaluations of the features of sagittal spinopelvic alignment have received increasing attention over the last 30 years. For clinical reasons and in the treatment of spinopelvic disorders, this is crucial information. Consequently, there is a considerable degree of harmony between spinopelvic features [4,5].

Lumbar disc herniation, disc degeneration, and spondylolisthesis are illnesses that are closely linked to variances in individuals' spinopelvic parameters. In addition, the exact criteria for determining if surgical intervention is required to treat lumbar disc prolapse has been discussed at length in the literature [6]; it can be treated non-invasively using medicine, physiotherapy, targeted exercises, epidural steroid injections, and platelet-rich plasma (PRP) injections [7].

PRP is obtained through the collection of the patient's own blood that has platelet levels higher than the normal physiological level, after which the process of centrifugation is used to separate the liquid and solid components of blood [8,9]. PRP therapy has recently gained interest as a safe and non-surgical treatment for osteoarthritis and musculoskeletal repair [10]. Interleukin-1 receptor antagonists, transforming growth factor- β1, platelet-derived growth factor, and insulin-like growth factor-1 are some cytokines and growth factors that are present in PRP therapy. Because of the utilization of autologous blood, PRP therapy is associated with a decreased likelihood of immunogenic reactions, adverse effects, and surgical site infections [7]. There is insufficient data showing its efficacy in treating intervertebral disc degeneration and low back pain. This article aims to provide insight into the utilization of transforaminal PRP injection as a treatment for discogenic low back pain in individuals with modified spinopelvic parameters. We evaluated the decrease in pain and improvement in functional outcomes of patients with modified spinopelvic parameters following transforaminal autologous PRP injection intervention.

## Materials and methods

Study setting and population

The observational study was conducted in the orthopaedic department of RL Jalappa Hospital and Research Centre, which is affiliated with Sri Devaraj Urs Medical College and Sri Devaraj Urs Academy of Higher Education and Research (SDUAHER). The study took place from September 2022 to August 2023 and included the patients who were admitted to the orthopaedic ward from the emergency medicine and outpatient department at this institute and diagnosed with lumbar disc prolapse. 

Inclusion criteria

The study included those patients who were over 18 years of age with a minimum of six months of low back pain history and single-level disc prolapse as well as those currently experiencing leg pain and chronic pain that did not respond to conservative treatment options, such as physiotherapy and non-opioid medications. Additionally, patients diagnosed with lumbar facet joint degenerative abnormalities on lumbar spine imaging (preferably on MRI) and those who willingly offered written informed consent and actively engaged in outcome assessments were also included. 

Exclusion criteria

The study excluded patients with a prior history of spinal surgery or a record of receiving epidural injections, progressive neurological decline, compression of the cauda equina, problems related to blood clotting abnormalities, concurrent cervical spinal cord dysfunction, and systemic disorders affecting the bones and joints. Other contraindications were hypersensitivity to local anaesthetics, pregnancy or breastfeeding, lumbar spinal stenosis or spinal instability, and vertebral fractures.

Sample size

The sample size was selected by considering a variance difference of 13.455 and an expected minimum mean difference of five units. By considering a dropout rate of 10%, the sample size was determined to be 64 individuals. The precise value of the standard deviation was obtained from a research study conducted by Wongjarupong et al. in Thailand in 2023 [6]. To enhance the reliability of our study, we have included 100 individuals, based on the above calculations.

Sample technique

Patients who met the specific eligibility requirements and showed their willingness to take part in the trial were eligible for participation. More precisely, the first 100 patients who met these specific requirements and were admitted to this institution's orthopaedics ward were included in the study. These patients were selected from both the emergency medical department and the outpatient department. To make the recruiting process more efficient, an easy sampling technique was implemented to pick participants.

Ethics approval

Upon obtaining clearance from the Institutional Ethical Committee of SDUMC (clearance number: SDUMC/KLR/IEC/583/2023-24), eligible patients who expressed their willingness to take part and met the specific inclusion criteria were enrolled in the trial after providing written informed consent.

Data collection procedure

Every patient underwent a thorough evaluation that included a full medical history, clinical examination, and imaging of the lumbosacral spine from both front and side views, to confirm their eligibility based on certain criteria. After obtaining the patient's consent and confirming their fitness for surgery, a PRP injection was administered. The injection technique followed the standardized protocol and was performed by an experienced spine surgeon in collaboration with orthopaedic residents. Prior to the PRP injection, the intensity of pain and the individual's functional level were evaluated. These assessments were again conducted one month and six months following the PRP injection. The assessment of spinopelvic parameters was conducted using a computer-based system. The specific measurement techniques include [3,11,12]:

Pelvic incidence (PI) is defined as the angle created by a vertical line drawn from the middle of the sacral 1st vertebra's endplate and the place where this line intersects with the axis of the femoral head.

The sacral slope (SS) is determined by measuring the angle formed between the line that runs tangent to the upper endplate of the sacrum 1 and a horizontal plane.

Pelvic tilt (PT) is the measurement of the angle formed by a vertical line passing through the centre of the femoral head and another vertical line connecting this point to the midpoint of the sacral endplate.

Lumbar lordosis (LL) is measured using the Cobb method, which quantifies the angle that exists between the lowest endplate of the T12 vertebra and the higher endplate of the first sacral vertebra.

PRP preparation 

The PRP was made using the conventional two-step centrifugation technique. We collected 50-75 mL of peripheral blood samples under sterile circumstances (based on the number of treatment tiers) using a blood bag. Next, we initially centrifuged the sample using a light spin at 2,630 revolutions per minute for three minutes at room temperature to obtain the entire serum supernatant and a tiny portion of the sub-natant erythrocyte. We performed a second centrifugation at 1500 RPM for another 15 minutes on the serum supernatant to extract a portion of the platelet-poor plasma. Ultimately, we extracted and prepared 10-15 ml of autologous PRP for injection. We conducted tests on each enrolled patient's complete blood count (CBC) in native peripheral blood prior to treatment and platelet concentration in PRP following standard centrifugations to confirm that the concentration of platelets in PRP was approximately four to five times greater than that in native peripheral blood.

PRP injection procedure

Prior to the treatment, the specified injection site was meticulously cleansed with surgical spirit three separate times. Subjects had to lie in a prone position. The surface was then protected with a sterile covering to ensure that aseptic conditions were maintained. For guidance, a transforaminal technique utilizing C-arm fluoroscopy was implemented through a region known as Kambin's triangle. This particular method was selected in order to mitigate the potential danger of nerve injury. Initially, a local anaesthetic, specifically 3 cc of 2% prilocaine, was administered to the epidermis and subcutaneous layers. Consequently, the slender point of a 22-gauge spinal needle was gradually advanced toward the six o'clock orientation of the pedicle with the assistance of intermittent fluoroscopic guidance.

In order to verify that the probe was positioned accurately within the sub-pedicular region, a lateral view was employed. Based on the observations, it was determined that 2-3 mL of PRP should be administered.

In order to instantly identify and resolve any possible adverse effects, patients were observed for a period of one hour following the procedure. Participants were later discharged with directions to return for a second appointment after the observation period.

Study tools

The visual analogue scale (VAS) is a linear measurement tool that uses endpoints to represent absolute boundaries. Scoring is accomplished by manually marking a 10-cm line that symbolizes a spectrum ranging from "absence of pain" to "most severe pain." The patient is instructed to show the intensity of his discomfort by marking a point on the line that connects the two ends. The subject's pain is determined by the distance between the absence of pain and the mark [13].

The Oswestry Disability Index (ODI) is the predominant assessment tool used in hospitals to measure the impact of low back pain. It is an instrument for self-administration divided into ten questions aimed at measuring the constraints of various activities of daily living. Each component is graded on a 0-5 scale, with 5 reflecting the most disability [14].

Statistical analysis

All data were entered in Excel (Microsoft Corp., Redmond, WA) and analyzed in SPSS Statistics version 26.0 (IBM Corp., Armonk, NY). The categorical variables were represented using frequency and percentage. The mean and standard deviation were used to express all the quantitative variables. The association between two category variables was assessed using a chi-square test. An independent t-test was used to examine the pain score and ODI score in patients with increasing and decreased spino-pelvic parameters. The paired t-test was used to examine the difference in pain reduction and functional outcome before and after PRP injection in patients with altered spino-pelvic parameters. If the p-value was less than 0.05, we regarded it as statistically significant.

## Results

The average age of the individuals involved in the study was 41.82 ± 5.0 years, and 55% of them were male. Our observations show that 39 (39%) research samples exhibit elevated spinopelvic parameter angles. Table 1 displays the fundamental attributes of the research population.

**Table 1:** Basic characteristics of the study population

Variable		Frequency (Percent)
Age in years (mean and standard deviation)		41.82 ± 5.0
Gender	Male	55 (55%)
	Female	45 (45%)
Spinopelvic parameters	Increased angle	39 (39%)
	Decreased angle	61 (61%)

Table 2 shows the mean and standard deviation values for all spinopelvic parameters. All the parameters were observed to be within their normal range.

**Table 2:** Mean values of all spino-pelvic parameters

Spino-pelvic parameters	Increased angle of SPP (n=39)		Reduced angle of SPP (n=61)	
	Mean	Std. Deviation	Mean	Std. Deviation
PI pre-injection	52.549	1.3751	44.466	2.1183
PI at 1 month	52.546	1.4164	44.411	2.1064
PI at 6 months	52.577	1.4111	44.462	2.112
PT pre-injection	14.144	0.5821	13.048	0.67
PT at 1 month	14.187	0.625	13.046	0.6574
PT at 6 months	14.133	0.5913	13.093	0.6468
SS pre-injection	38.454	1.7741	31.418	1.8261
SS at 1 month	38.377	1.8281	31.367	1.8305
SS at 6 months	38.474	1.8225	31.336	1.8779
LL pre-injection	54.508	0.9059	46.941	2.1866
LL at 1 month	54.736	0.8907	46.89	2.1858
LL at 6 months	54.882	0.907	47.02	2.2899

The spinopelvic characteristics were shown to be independent of age (p=0.541) but were significantly influenced by gender (p=0.05). The independent t-test revealed a statistically significant difference in the mean scores of back pains, leg pain, and ODI scores between patients with increased and decreased angles of spinopelvic parameters before and after injection (p=0.0001). Table 3 displays a comparison of age, gender, severity of pain, and functional status between patients with increased and decreased angles of spinopelvic parameters before and after PRP injections.

**Table 3:** Comparison of age, gender, severity of pain, and functional status between patients with increased and decreased angles of spinopelvic parameters before and after PRP injections ODI: Oswestry Disability Index; PRP: platelet-rich plasma; SPP: spinopelvic parameters The chi-square test^#^ and the independent t-test^*^ were used; significance was set at a p-value <0.05

Variables	Increased angle of SPP (n=39), represented as mean values	Reduced angle of SPP (n=61), represented as mean values	Mean difference	P-value
Age in years	42.21	41.57	0.64	0.541*
Gender, male/female (frequency)	17/22	38/23	-	0.05^#^
Back pain pre-injection	7.41	6.82	0.59	0.0001*
Back pain at 1 month	4.51	2.79	1.72	0.0001*
Back pain at 6 months	6.13	2.72	3.41	0.0001*
Leg pain pre-injection	7	6.44	0.56	0.0001*
Leg pain at 1 month	4.67	2.92	1.75	0.0001*
Leg pain at 6 months	5.95	2.80	3.15	0.0001*
ODI pre-injection	23	17.41	5.59	0.0001*
ODI at 1 month	16.41	8.33	8.08	0.0001*
ODI at 6 months	17.08	8.1	8.97	0.0001*

Among the patients with higher spinopelvic parameter angles, the intensity of back pain and leg discomfort decreased considerably after receiving PRP injections at one month (p=0.0001) and six months (p=0.0001) compared to before the injections. Patients who had an elevated angle experienced a substantial reduction in functional impairment after receiving PRP injections at both one month (p=0.0001) and six months (p=0.0001) compared to before the injections. Patients with higher spinopelvic parameter angles experienced a significant reduction in the severity of back pain, leg pain, and functional disability after receiving PRP injections at one month (p=0.0001). However, at the six-month follow-up, these patients exhibited recurring symptoms and worsening back pain, leg pain, and functional disability compared to the one-month follow-up. The severity of pain and functional status of patients with increased spinopelvic parameter angles were compared before and after PRP injections. The results can be found in Table 4.

**Table 4:** Comparison of severity of pain and functional status among patients with increased SPP angles before and after PRP injections ODI: Oswestry Disability Index; PRP: platelet-rich plasma; SPP: spinopelvic parameter *The paired T-test was used; significance was set at a p-value <0.05

Pairs	Period of assessment	Mean	Std. deviation	Mean difference	P-value
Pair 1	Back pain pre-injection	7.41	0.498	2.897	0.0001*
	Back pain at 1 month	4.51	0.506		
Pair 2	Back pain pre-injection	7.41	0.498	1.282	0.0001*
	Back pain at six months	6.13	0.767		
Pair 3	Back pain at one month	4.51	0.506	-1.615	0.0001*
	Back pain at six months	6.13	0.767		
Pair 4	Leg pain pre-injection	7.00	0.000	2.333	0.0001*
	Leg pain at 1 month	4.67	0.478		
Pair 5	Leg pain pre-injection	7.00	0.000	1.051	0.0001*
	Leg pain at 6 months	5.95	0.686		
Pair 6	Leg pain at 1 month	4.67	0.478	-1.282	0.006*
	Leg pain at 6 months	5.95	0.686		
Pair 7	ODI pre-injection	23.00	0.918	6.59	0.0001*
	ODI at 1 month	16.41	0.966		
Pair 8	ODI pre-injection	23.00	0.918	5.923	0.0001*
	ODI at 6 months	17.08	1.133		
Pair 9	ODI at 1 month	16.41	0.966	-0.667	0.0001*
	ODI at 6 months	17.08	1.133		

Patients with decreased spinopelvic parameter angles saw a significant reduction in the severity of back pain and leg discomfort after receiving PRP injections. This reduction was observed at both one (p=0.0001) and six months (p=0.0001) post-injections, compared to their pre-injection levels. Patients with a decreased angle experienced a substantial reduction in functional impairment after receiving PRP injections at both one (p=0.0001) and six months (p=0.0001) compared to before the injections. The severity of pain and functional status of patients with decreased angles of spinopelvic parameters before and after PRP injections are compared in Table 5. Over the course of the six-month follow-up period, we did not encounter any complications in our investigation. 

**Table 5:** Comparison of severity of pain and functional status among patients with decreased SPP angles before and after PRP injections ODI: Oswestry Disability Index; PRP: platelet-rich plasma; SPP: spinopelvic parameter *The paired T-test was used; significance was set at a p-value <0.05

Pairs	Period of assessment	Mean	Std. Deviation	Mean difference	P-value
Pair 1	Back pain pre-injection	6.82	0.671	4.033	0.0001*
	Back pain at 1 month	2.79	0.451		
Pair 2	Back pain pre-injection	6.82	0.671	4.098	0.0001*
	Back pain at 6 months	2.72	0.609		
Pair 3	Back pain at 1 month	2.79	0.451	0.066	0.209
	Back pain at 6 months	2.72	0.609		
Pair 4	Leg pain pre-injection	6.44	0.696	3.525	0.0001*
	Leg pain at 1 month	2.92	0.666		
Pair 5	Leg pain pre-injection	6.44	0.696	3.639	0.0001*
	Leg pain at 6 months	2.8	0.726		
Pair 6	Leg pain at 1 month	2.92	0.666	0.115	0.07
	Leg pain at 6 months	2.8	0.726		
Pair 7	ODI pre-injection	17.41	2.171	9.082	0.0001*
	ODI at 1 month	8.33	2.688		
Pair 8	ODI pre-injection	17.41	2.171	9.311	0.0001*
	ODI at 6 months	8.1	2.675		
Pair 9	ODI at 1 month	8.33	2.688	0.23	0.009*
	ODI at 6 months	8.1	2.675		

**Figure 1:**
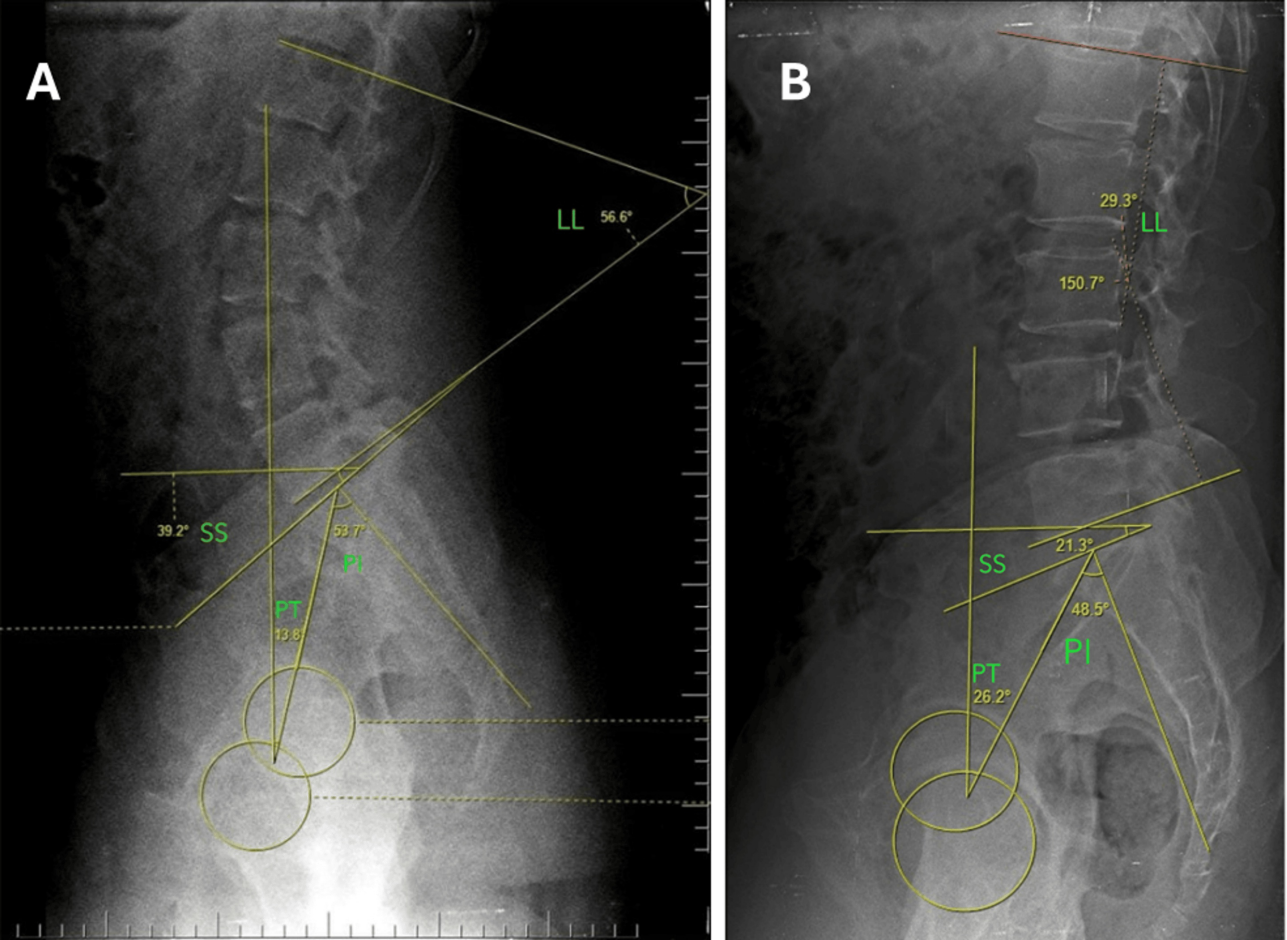
Measurement of assessed spinopelvic parameters A. The patient with higher spinopelvic parameters had the following measurements: LL; 56.6º, PT; 13.8º; PI, 53.7º; SS, 39.2 B. The patient with lower spinopelvic parameters had the following measurements: LL, 29.3º; PT, 26.2º; PI, 48.5º; SS, 21.3 LL: lumbar lordosis; PT: pelvic tilt; PI: pelvic incidence; SS: sacral slope

Figure 1 shows the measurements of spinopelvic parameter angles assessed in two patients; (A) the first patient had increased spinopelvic parameter angles and the other patient (B) had decreased spinopelvic parameter angles. 

## Discussion

PI, PT, and SS are commonly used measurements to assess pelvic structure and alignment, providing a comprehensive understanding of the pelvis's orientation and curvature. The PI, which remains consistent and unique to each person, serves as a morphological measure to characterize the sacro-pelvis. Duval-Beaupère et al. proposed a parameter known as the sacral pelvic angle, which is defined as “the angle between the perpendicular line to the upper sacral endplate and the line connecting the midpoint of the upper sacral endplate to the hip axis [15].” The link between lumbar spine disc diseases and degeneration has been emphasized by multiple writers, particularly in relation to the spinopelvic structure [16]. The determining factor lies in the angle of pelvic incidence (PI). Singh et al. have reported that the mean value of PI in the Indian population is 48.52 ± 8.99 [17]. In contrast to the PI, the PT, and SS focus on evaluating the alignment of the sacro-pelvis from a sagittal plane perspective.

The measurement of SS represents the angle between the sacral endplate and a horizontal reference line. Conversely, PT is the angle measured between a vertical reference line and a line that connects the midpoint of the sacral endplate to the hip axis. According to research conducted on the Indian population, it has been determined that the average values for SS and PT are 39.14 ± 7.05 and 9.30 ± 7.16, respectively. The sum of PT and SS is equal to the value of PI, denoted by the equation PI = PT + SS [17].

In India, Poonia et al. conducted a hospital-based cross-sectional study in 2020 that involved 60 cases of prolapsed disc [3]. The average age in this study was 39.27 years. The level L5-S1 was the most frequently observed. The mean values for SS, PT, PI, and LL were 37.78°, 13.52°, 51.33°, and 41.01°, respectively. The study found a connection of disc diseases at the L1-L2, L2-L3, and L4-L5 levels to PT, PI, and LL as well as a connection of disc disease at the L5-S1 level to PT and LL [3].

The distribution of load along the lumbar spine is disrupted when there is a deviation from the normal range of LL, which expedites the deterioration of the intervertebral discs. The research conducted by Keorochana et al. revealed that variations in the sagittal plane alignment of the spine and pelvis can influence the load and movement of the spine, possibly leading to the degeneration of specific segments. Furthermore, these alterations in alignment can cause changes in weight distribution and the distribution of spinal disc degeneration at different levels [18]. Hence, the assessment of sagittal balance has become essential in addressing lumbar degenerative conditions.

The present study shows that in patients with increased spinopelvic parameter angles, the severity of back pain, leg pain, and functional disability has reduced significantly after PRP injections at one month (p=0.0001); however, at the six-month follow-up, patients presented with recurring symptoms and worsening back pain, leg pain, and functional disability compared to the one-month follow-up. On the other hand, among the patients with lower angles in assessed spinopelvic parameter angles, the severity of back pain, leg pain, and functional disability has reduced significantly after PRP injections at one month (p=0.0001) and six months (p=0.0001) compared to the pre-injection period. Given that PRP contains both pro- and anti-inflammatory mediators, this pattern may have a biological reason. Regenerative medicine using PRP has gained popularity. Particularly in degenerative tissues, the elevated concentrations of secretory proteins and autologous growth factors offered by concentrated platelets may hasten the healing process [19]. When triggered, anti-inflammatory cytokines ease pain and improve healing by changing the microenvironment [20]. There is enough data from animal studies to conclude that PRP stimulates cell proliferation and extracellular matrix regeneration [21].

In 2023, Le et al. conducted a prospective research study in Vietnam, focusing on 25 patients diagnosed with lumbar disc herniation. Using fluoroscopy, the doctors administered a 4 ml injection of autologous platelet-rich plasma, delivering it directly to the affected nerve root through trans-foraminal epidural injection [22]. The patients' functional status and VAS scores showed a significant improvement following transforaminal injections of autologous PRP. Throughout the 12-month post-treatment period, there were no adverse reactions reported, and the advantages remained evident [22].

Although patients showed significant improvement in back pain, leg pain, and functional outcome for the one-month follow-up compared to pre-injection irrespective of the measured spinopelvic parameters, patients with decreased angles in spinopelvic parameters showed better sustained functional outcomes at the six-month follow-up whereas patients with increased spinopelvic parameter angles presented with recurring symptoms and worsening back pain, leg pain, and functional disability at the six-month follow-up. Contrary to expectations, our analysis revealed no significant changes in spinopelvic parameters, as measured by specific angles, i.e., PI, SS, PT, and LL, during follow-up assessments in individual subjects. However, a notable observation was that patients with lower-range spinopelvic parameters demonstrated a longer symptom-free period compared to those with higher-range parameters. This suggests that while spinopelvic parameters may not undergo significant changes following PRP treatment, their baseline values may influence treatment outcomes. Specifically, patients with more optimal spinopelvic alignment (lower range parameters) tend to experience better functional outcomes, as evidenced by prolonged symptom-free periods. Further research is warranted to explore the underlying mechanisms and clinical implications of this finding.

The study revealed that a significant number of patients experienced an enhanced quality of life as they regained their functional status, allowing them to resume their daily activities. The effectiveness of this method was equal to that of standard steroid injections. By delivering concentrated growth factors and cytokines, this innovation sought to optimize the body's inherent healing abilities. Studies have shown that these growth factors can effectively enhance cell proliferation, promote the formation of new blood vessels, and increase the production of proteins in the extracellular matrix [23-25]. Taking these findings into account, it is strongly recommended to conduct further research on the potential benefits of using autologous PRP instead of epidural steroids. When it comes to easing pain caused by lumbar disc herniation, PRP emerges as a highly effective biological treatment option. Over the course of the six-month follow-up period, we did not encounter any significant drawbacks or complications in our investigation. The simultaneous occurrence of infections and hematomas is widely recognized because of epidural steroid injection [26,27]. By harnessing the antimicrobial proteins found in the patient's own blood, autologous PRP becomes a safer choice, significantly decreasing the chances of infection and allergic reactions [28].

Limitations

The limitation of our study was the limited sample size. To conduct the study within the specified time frame, we have chosen a smaller sample size that is appropriate for the number of patients in our outpatient department. Despite the limited sample size, our study is nevertheless significant as it will be a basis for future research; however, we cannot apply our findings to the entire population. In addition, our study excluded asymptomatic patients whose lumbar disc prolapse was discovered incidentally at our outpatient clinic while seeking treatment for another medical condition. Because our study was conducted at a single centre and had a small sample size, the statistical power of the study is inadequate, which limits the external validity. To further validate our results, it is necessary to conduct additional multicentre studies with larger sample sizes.

## Conclusions

Transforaminal PRP injections show encouraging outcomes in the treatment of lumbar disc prolapse cases accompanied by radicular discomfort. Individuals exhibiting elevated spinopelvic parameter levels experienced a diminished degree of amelioration in both back pain, leg pain, and functional results during an extended period of observation. The angles of these spinopelvic parameters significantly impact the intensity of back pain and functional impairment. Consequently, it plays a crucial role in determining the functional outcome of patients with lumbar disc prolapse. This study examines the correlation between spinopelvic parameters and the functional result in patients who underwent transforaminal PRP injections for lumbar disc prolapse. However, further prospective controlled trials are required to assess the impact of biomechanics and potential alterations in lumbar disc herniation on the efficacy of transforaminal PRP treatment.

## References

[1] Mirzashahi B, Hajializade M, Abdolahi Kordkandi S (2023). Spinopelvic parameters as risk factors of nonspecific low back pain: a case-control study. Med J Islam Repub Iran.

[2] Yazici Sacaklidir G, Sencan S, Sacaklidir R, Gunduz OH (2021). The effect of spinopelvic parameters on transforaminal epidural steroid injection treatment success in lumbar disc herniation. Int J Clin Pract.

[3] Poonia A, Lodha S, Sharma NC (2020). Evaluation of spinopelvic parameters in lumbar prolapsed intervertebral disc. Indian J Radiol Imaging.

[4] Vrtovec T, Janssen MM, Likar B, Castelein RM, Viergever MA, Pernuš F (2012). A review of methods for evaluating the quantitative parameters of sagittal pelvic alignment. Spine J.

[5] Boulay C, Tardieu C, Hecquet J (2006). Sagittal alignment of spine and pelvis regulated by pelvic incidence: standard values and prediction of lordosis. Eur Spine J.

[6] Wongjarupong A, Pairuchvej S, Laohapornsvan P, Kotheeranurak V, Jitpakdee K, Yeekian C, Chanplakorn P (2023). "Platelet-Rich Plasma" epidural injection an emerging strategy in lumbar disc herniation: a Randomized Controlled Trial. BMC Musculoskelet Disord.

[7] Datt R, Jain G, Krishna A, Vijayakumar V, Tank S (2023). Association of various spinopelvic parameters and the quality of life in those with degenerative lumbar scoliosis in the Indian population. Cureus.

[8] Hall MP, Band PA, Meislin RJ, Jazrawi LM, Cardone DA (2009). Platelet-rich plasma: current concepts and application in sports medicine. J Am Acad Orthop Surg.

[9] Hsu WK, Mishra A, Rodeo SR (2013). Platelet-rich plasma in orthopaedic applications: evidence-based recommendations for treatment. J Am Acad Orthop Surg.

[10] Mohammed S, Yu J (2018). Platelet-rich plasma injections: an emerging therapy for chronic discogenic low back pain. J Spine Surg.

[11] Gao A, Wang Y, Yu M, Wei F, Jiang L, Liu Z, Liu X (2020). Association between radiographic spinopelvic parameters and health-related quality of life in de novo degenerative lumbar scoliosis and concomitant lumbar spinal stenosis. Spine (Phila Pa 1976).

[12] Borkar SA, Sharma R, Mansoori N, Sinha S, Kale SS (2019). Spinopelvic parameters in patients with lumbar degenerative disc disease, spondylolisthesis, and failed back syndrome: Comparison vis-à-vis normal asymptomatic population and treatment implications. J Craniovertebr Junction Spine.

[13] Haefeli M, Elfering A (2006). Pain assessment. Eur Spine J.

[14] Mehra A, Baker D, Disney S, Pynsent PB (2008). Oswestry Disability Index scoring made easy. Ann R Coll Surg Engl.

[15] Duval-Beaupère G, Schmidt C, Cosson P (1992). A Barycentremetric study of the sagittal shape of spine and pelvis: the conditions required for an economic standing position. Ann Biomed Eng.

[16] Roussouly P, Berthonnaud E, Dimnet J (2003). Geometrical and mechanical analysis of lumbar lordosis in an asymptomatic population: proposed classification [article in French]. Rev Chir Orthop Reparatrice Appar Mot.

[17] Singh R, Yadav SK, Sood S, Yadav RK, Rohilla R (2018). Spino-pelvic radiological parameters in normal Indian population. SICOT J.

[18] Keorochana G, Taghavi CE, Lee KB, Yoo JH, Liao JC, Fei Z, Wang JC (2011). Effect of sagittal alignment on kinematic changes and degree of disc degeneration in the lumbar spine: an analysis using positional MRI. Spine (Phila Pa 1976).

[19] Arnoczky SP, Sheibani-Rad S (2013). The basic science of platelet-rich plasma (PRP): what clinicians need to know. Sports Med Arthrosc Rev.

[20] Anitua E, Prado R, Orive G (2017). Allogeneic platelet-rich plasma: at the dawn of an off-the-shelf therapy?. Trends Biotechnol.

[21] Gupta A, Chhabra HS, Singh V, Nagarjuna D (2024). Lumbar transforaminal injection of steroids versus platelet-rich plasma for prolapse lumbar intervertebral disc with radiculopathy: a randomized double-blind controlled pilot study. Asian Spine J.

[22] Le VT, Nguyen Dao LT, Nguyen AM (2023). Transforaminal injection of autologous platelet-rich plasma for lumbar disc herniation: A single-center prospective study in Vietnam. Asian J Surg.

[23] Mei-Dan O, Carmont MR, Laver L, Mann G, Maffulli N, Nyska M (2012). Platelet-rich plasma or hyaluronate in the management of osteochondral lesions of the talus. Am J Sports Med.

[24] Ficek K, Kamiński T, Wach E, Cholewiński J, Cięszczyk P (2011). Application of platelet rich plasma in sports medicine. J Hum Kinet.

[25] Andia I, Sánchez M, Maffulli N (2012). Basic science: molecular and biological aspects of platelet-rich plasma therapies. Oper Tech Orthop.

[26] Pinto RZ, Maher CG, Ferreira ML (2012). Epidural corticosteroid injections in the management of sciatica: a systematic review and meta-analysis. Ann Intern Med.

[27] Friedly JL, Comstock BA, Turner JA (2014). A randomized trial of epidural glucocorticoid injections for spinal stenosis. N Engl J Med.

[28] Centeno C, Markle J, Dodson E, Stemper I, Hyzy M, Williams C, Freeman M (2017). The use of lumbar epidural injection of platelet lysate for treatment of radicular pain. J Exp Orthop.

